# Comparative transcriptomic analysis reveals the mechanistic basis of *Pib*-mediated broad spectrum resistance against *Magnaporthe oryzae*

**DOI:** 10.1007/s10142-020-00752-x

**Published:** 2020-09-07

**Authors:** Jiehua Qiu, Feifei Lu, Meng Xiong, Shuai Meng, Xianglin Shen, Yanjun Kou

**Affiliations:** 1 State Key Laboratory of Rice Biology, China National Rice Research Institute, Hangzhou, 311400 China; 2 Key Laboratory of Three Gorges Regional Plant Genetics & Germplasm Enhancement (CTGU)/Biotechnology Research Center, China Three Gorges University, Yichang, 443000 China

**Keywords:** *Magnaporthe oryzae*, Resistant near isogenic line, RNA-seq, Broad-spectrum resistance, *Pib*, Redox

## Abstract

Rice blast, caused by the fungus *Magnaporthe oryzae*, is a highly damaging disease. Introducing genes, which confer a broad spectrum resistance to the disease, such as *Pib*, makes an important contribution to protecting rice production. However, little is known regarding the mechanistic basis of the products of such genes. In this study, transcriptome of the cultivar Lijiangxintuanheigu (LTH) and its monogenic IRBLb-B which harbors *Pib* treated with *M. oryzae* were compared*.* Among the many genes responding transcriptionally to infection were some encoding products involved in the metabolism of ROS (reactive oxygen species), in jasmonate (JA) metabolism, and WRKY transcription factors, receptor kinases, and resistance response signal modulation. The down-regulation of genes encoding peroxiredoxin and glutathione S transferases implied that the redox homeostasis is essential for the expression of *Pib*-mediated resistance. The up-regulation of seven disease resistance-related genes, including three encoding a NBS-LRR protein, indicated that disease resistance-related genes are likely tend to support the expression of *Pib* resistance. These data revealed that potential candidate genes and transcriptional reprogramming were involved in *Pib*-mediated resistance mechanisms.

**Electronic supplementary material:**

The online version of this article (10.1007/s10142-020-00752-x) contains supplementary material, which is available to authorized users.

## Introduction

Rice is a staple food for half of the world’s population. Like all crops, its production is constrained by a number of both biotic and abiotic stresses. Among the various stresses, rice blast, which is caused by fungal pathogen *Magnaporthe oryzae*, is the most devastating disease in rice production. It occurs almost in all rice planting areas, generally resulting in rice yield loss of 10 to 30% annually (Dean et al. 2012). An increase in understanding the molecular mechanisms of the rice–*M. oryzae* interaction may be useful to develop novel strategies for blast control.

To resist pathogen invasion, plants have developed a complex system of defense against microbial pathogens such as *M. oryzae*: the first layer involves pattern recognition receptors (PRRs), which trigger immunity after they recognize the presence of the pathogen-associated molecular patterns (PAMPs); the second layer is effectors trigger immunity (ETI), which relies on resistance (R) proteins recognize pathogen avirulence factors, after which a hypersensitive response (HR) is rapidly mounted (Sakulkoo et al. 2018). The latter mode of resistance has proven to be a highly effective means of endowing rice cultivars with immunity against blast (Fernandez and Orth 2018). To date, more than 100 rice *R* genes have been mapped and 35 have been isolated (Wang et al. 2017). Most of these *R* genes have been found to encode proteins belonging to the nucleotide-binding site leucine-rich repeat (NBS-LRR) family; one of these is *Pib*, carriers of which are resistant to a lot of the *M. oryzae* strains found in Japan and some of those found in China. As a result, *Pib* is considered to give broad spectrum resistance (Ramkumar et al. 2015; Wang et al. 1999; Zhang et al. 2018b), and so has been used by breeders (Fjellstrom et al. 2004; Koide et al. 2010; Singh et al. 2015; Tanweer et al. 2015). Until now, it is unclear how *Pib*-mediated resistance is affected.

An effective way of gauging the effect of a single gene is to base comparisons on pairs of so-called near-isogenic lines (NILs), which, as a result of the back-crossing procedure used for their construction, differ from one another with respect to the target gene and just a small number of other genes (Fukuoka et al. 2015; Yuan et al. 2017). Thus, for example, transcriptomic comparisons between the Chinese cultivar LTH with its *Pi1* NIL IRBL18 and *Pi9* NIL IRBL22 have shown that a particular class of transcription factor is important for the expression of resistance determined by both resistance genes (Wei et al. 2013). Similarly, Jain et al. (2017) have been able to describe the set of genes which act to ensure the blast resistance determined by *Pi9* (Jain et al. 2017).

Here, similar strategy was directed at understanding the mechanistic basis of *Pib*-mediated resistance. In this study, the relevant NIL in a background of LTH was the line IRBLb-B; IRBLb-B (carrying *Pib*) is obtained from the *Pib* resistance donor parent BL1 crossed with recurrent parent LTH (Telebanco-Yanoria et al. 2010). The intention was to reveal the transcriptional differences between LTH and IRBLb-B when the plants were infected with *M. oryzae*. We expect that our results will improve the understanding of mechanisms of *Pib*-mediated broad-spectrum resistance against *M. oryzae* and facilitate breeding broad-spectrum resistance varieties.

## Materials and methods

### Plant materials and growing conditions

Grains of the *japonica* type rice cultivar LTH and IRBLb-B were obtained from the China National Rice Research Institute’s National Mid-term Genebank. The *oslox3*#KO was generated by CRISPR/cas9 system as described previously (Ma et al. 2015). The vector was transformed into XH11 (*Japonica*) callus using the Agrobacterium-mediated transformation method. The grains were surface-sterilized by immersion in 70% ethanol for 1 min, after which they were imbibed at 30 °C for 2 days and then allowed to germinate in moist towels at 37 °C for 24 h, transplanted the same development seedlings to sterile soil. The resulting seedlings were grown for 21 days in greenhouse soil providing a 14-h photoperiod and a constant temperature of 28 °C.

The presence of the effective allele at *Pib* in IRBLb-B and of the ineffective one in LTH was validated using a PCR assay based on, respectively, the primer pairs Pibdom F/R and Lys145 F/R, as described (Fjellstrom et al. 2004); the relevant primer sequences were given in Table S1.

### Fungal inoculation and RNA isolation

*M. oryzae* strain P131 was cultured on prune agar medium as described (Kou et al. 2017). Conidia were collected from the 7-day-old-culture by rinsing with sterile Milli-Q water and filtering through two layers of sterile miracloth (Millipore, Burlington, MA, USA). For the purpose of investigating the histological reaction of LTH or IRBLb-B plants to *M. oryzae* infection, *M. oryzae* conidial suspension of 10^5^ per mL was used to inoculate 5-cm lengths of leaf sheath cut from each of the two host genotypes, and the explants were maintained at 28 °C for either 26 h or 36 h. To generate samples for the purpose of RNA-seq analysis, 3-week-old seedlings were sprayed with a suspension of 10^6^ *M. oryzae* conidia per mL in water containing 0.01% w/v gelatin; the seedlings were kept in a dark humid chamber overnight at 22 °C, then transferred into a growth chamber providing a 16-h photoperiod and a constant temperature of 22 °C with 90% relative humidity. The spray used for mock inoculation was an aqueous solution of 0.01% w/v gelatin.

RNA was extracted from secondary leaves (three replicates per genotype) exposed to *M. oryzae* for 36 h; the harvested leaves were snap-frozen in liquid nitrogen, and their RNA was extracted using the TRIzol reagent according to the manufacturer’s (Invitrogen, Carlsbad, CA, USA) protocol. Genomic DNA was digested from the extracts using DNase I (TaKaRa, Dalian, China); the integrity of the RNA in the extracts was checked using a 2100 Bioanalyzer device (Agilent, Santa Clara, CA, USA) and its concentration quantified using a ND-2000 device (NanoDrop Technologies, www.thermofisher.com). The thresholds applied for accepting the samples for the RNA-seq procedure were OD_260/280_ 1.8–2.2, OD_260/230_ ≥ 2.0, RIN ≥ 6.5, and 28S:18S ≥ 1.0.

### Library preparation and sequencing

Each transcriptome library was constructed from an ~ 1-μg aliquot of total RNA and was processed following the protocol provided with a TruSeq^TM^ RNA sample preparation kit (Illumina, Inc., San Diego, CA). The libraries were size selected (200–300 bp) by agarose gel electrophoresis and were then PCR amplified over 15 cycles using Phusion DNA polymerase (NEB, Ipswich, MA, USA). After quantified by TBS380, paired-end sequencing was carried out using a HiSeq 4000 device (Illumina) operated by the Majorbio Genome Center (Shanghai, China).

### Differential transcription and functional enrichment analysis

The SeqPrep (github.com/jstjohn/SeqPrep) and Sickle (github.com/najoshi/sickle) software packages were used to control the quality of the raw reads, after which the reads were mapped onto the reference genome sequence of cv. Nipponbare (MSU release 7) using TopHat software (http://ccb.jhu.edu/software/tophat/downloads/) imposing a mismatch threshold of two nucleotides. To identify significant differential transcription between a pair of samples, transcript abundances were calculated using the “fragments per kilobase of exon per million mapped reads” method. A false discovery rate (FDR) threshold of 0.05, in conjunction with an absolute log_2_ fold change of 2, was imposed to call a gene significantly differentially expressed (hereafter referred to as SDEGs).

A functional enrichment analysis was applied to the full set of SDEGs, based on the Gene Ontology (GO) and the Kyoto Encyclopedia of Genes and Genomes (KEGG) databases taking advantage of the software tools Goatools (github.com/tanghaibao/Goatools) and KOBAS (kobas.cbi.pku.edu.cn/home.do). A heatmap was constructed using the cluster method, based on an in-house Perl script. The absolute log_2_ fold change data were used in Mapman software to get the Mapman view of biotic stress.

### Validation of differential transcription using quantitative real time PCR

Quantitative real time PCR (qRT-PCR) was used to validate differential transcription concluded from the RNA-seq data of twenty four of the SDEGs identified in the contrast IRBLb-B non-infected vs IRBLb-B infected with *M. oryzae*. Relevant PCR primers were designed using the GenScript Real-time PCR Primer Design tool (www.genscript.com/tools/real-time-pcr-tagman-primer-design-tool) and are shown in Table S1. The template for each qRT-PCR was a preparation of cDNA synthesized from total RNA by M-MLV reverse transcriptase (TaKaRa) and diluted 1:5 with RNAase free water to give a concentration of ~ 20 ng/μL. Each 10 μL reaction contained 5 μL TB Green Master (TaKaRa), 0.2 μL of each primer (10 mM), 2 μL templates, and 2.6 μL RNAase-free water. The sequence of the rice *Tubulin* gene (*LOC_Os03g13170*) was used as the reference (Liao et al. 2019). Relative transcript abundances were calculated using the 2^−ΔΔCT^ method (Livak and Schmittgen 2001).

### DAB and Trypan staining

The DAB (3, 3′-Diaminobenzidine) and Trypan were performed, as previously described (Zhu et al. 2013). The relative hydrogen peroxide level by ImageJ software (https://imagej.nih.gov/ij/), Briefly, the picture was reversed, and the coloring points turn to white. Then, the gray value of the area to be measured is determined. The relative hydrogen peroxide value was calculated according to the gray value. The LTH of 24 hpi was used as the reference.

## Results

### The response of LTH and IRBLb-B to *M. oryzae* infection

**Fig. 1:**
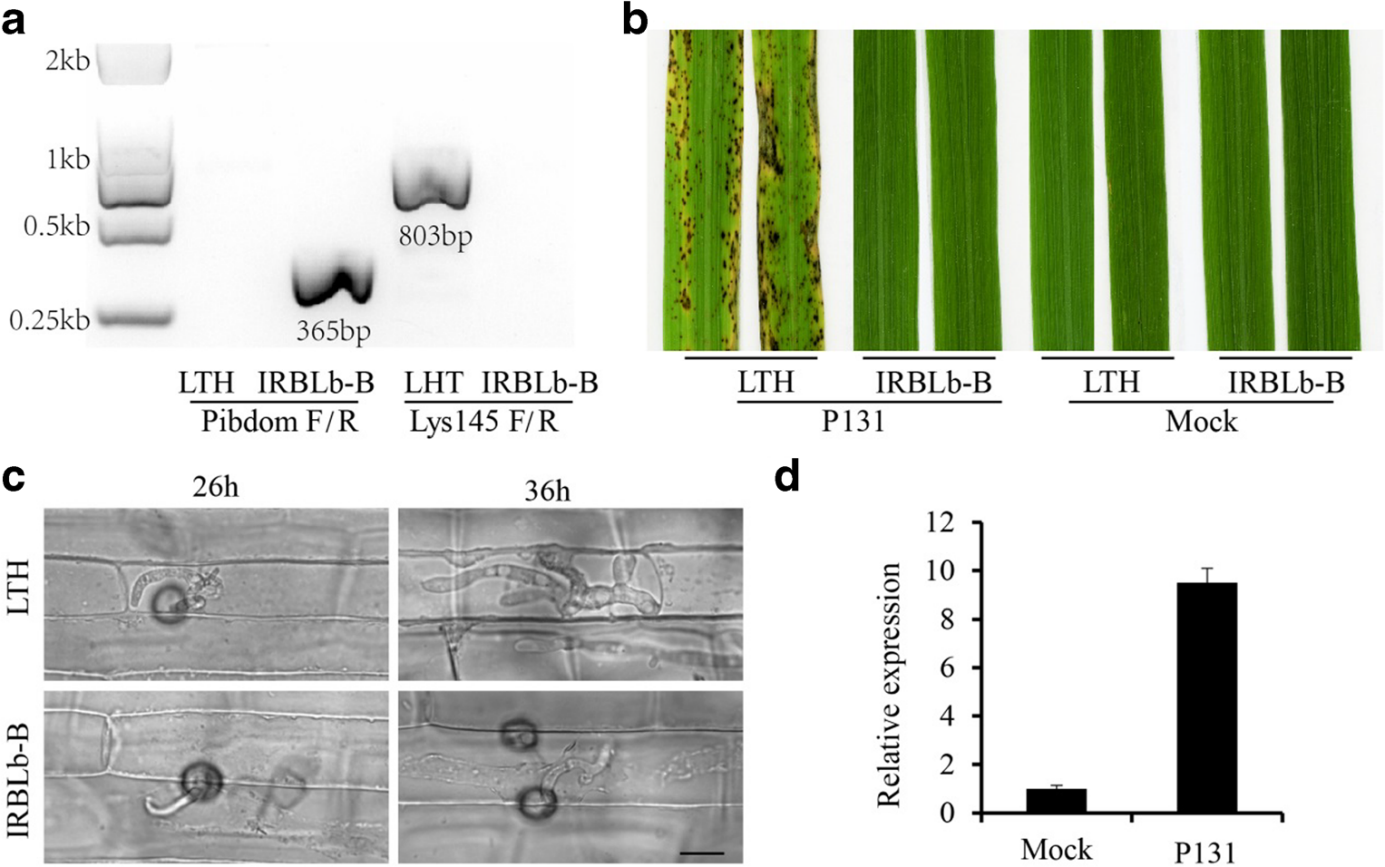
The response of LTH and IRBLb-B to infection by *M. oryzae* strain P131. **a** IRBLb-B harbors the resistance allele at *Pib*. The PCR product of PibdomF/R indicated that IRBLb-B contains the resistant *Pib* allele; The PCR product of Lys145F/R showed that LTH harbors the susceptible *Pib* allele. **b** The development of blast disease symptoms on the leaves of LTH and IRBLb-B with inoculation of *M. oryzae* strain p131 and Mock. The images were taken at 7 dpi (days post inoculation). **c** Micrographs of LTH and IRBLb-B leaf sheaths infected with *M. oryzae* strain P131 taken at 26 hpi and 36 hpi. Bar = 10 μm. At 36 hpi, the invasive hyphae extended to neighboring cells in the LTH, while the invasive hyphae were limited in the first invaded cell in the IRBLb-B. **d** The expression of *Pib* (*LOC_Os02g57310*) was induced almost nine times by the infection of *M. oryzae* at 36 hpi. The error bar means the standard deviation for triplicate assays

To verify the sequence identity of the *Pib* alleles present in IRBLb-B and LTH, the PCR assay along with DNA sequencing directed at *Pib* locus confirmed that IRBLb-B harbors the allele conferring blast resistance (Fig. 1a). When rice seedlings were inoculated with *M. oryzae* strain P131, LTH samples developed extensive lesions (Fig. 1b), while the IRBLb-B samples showed no reaction (Fig. 1b). We further investigated the penetrate pattern of *M. oryzae* in *Pib*-mediated incompatible interaction by rice sheath infection assay. *M. oryzae* appressoria were able to penetrate the sheath of both host genotypes by 26-hour post inoculation (hpi) (Fig. 1c). By 36 hpi, invasive hyphae had extended into the cells surrounding the penetration point in LTH, but in IRBLb-B, invasive hyphal growth was limited. In addition, the abundance of *Pib* transcript (*LOC_Os02g57310*) at 36 hpi was almost nine fold greater in the leaves of IRBLb-B inoculated with *M. oryzae* than mock (Fig. 1d). These results indicated that *Pib* expression was induced by rice blast infection and *Pib* resistance on fungal growth was detectible by 36 hpi.

### RNA-seq profiling of *M. oryzae*-inoculated LTH and IRBLb-B seedlings

**Fig. 2:**
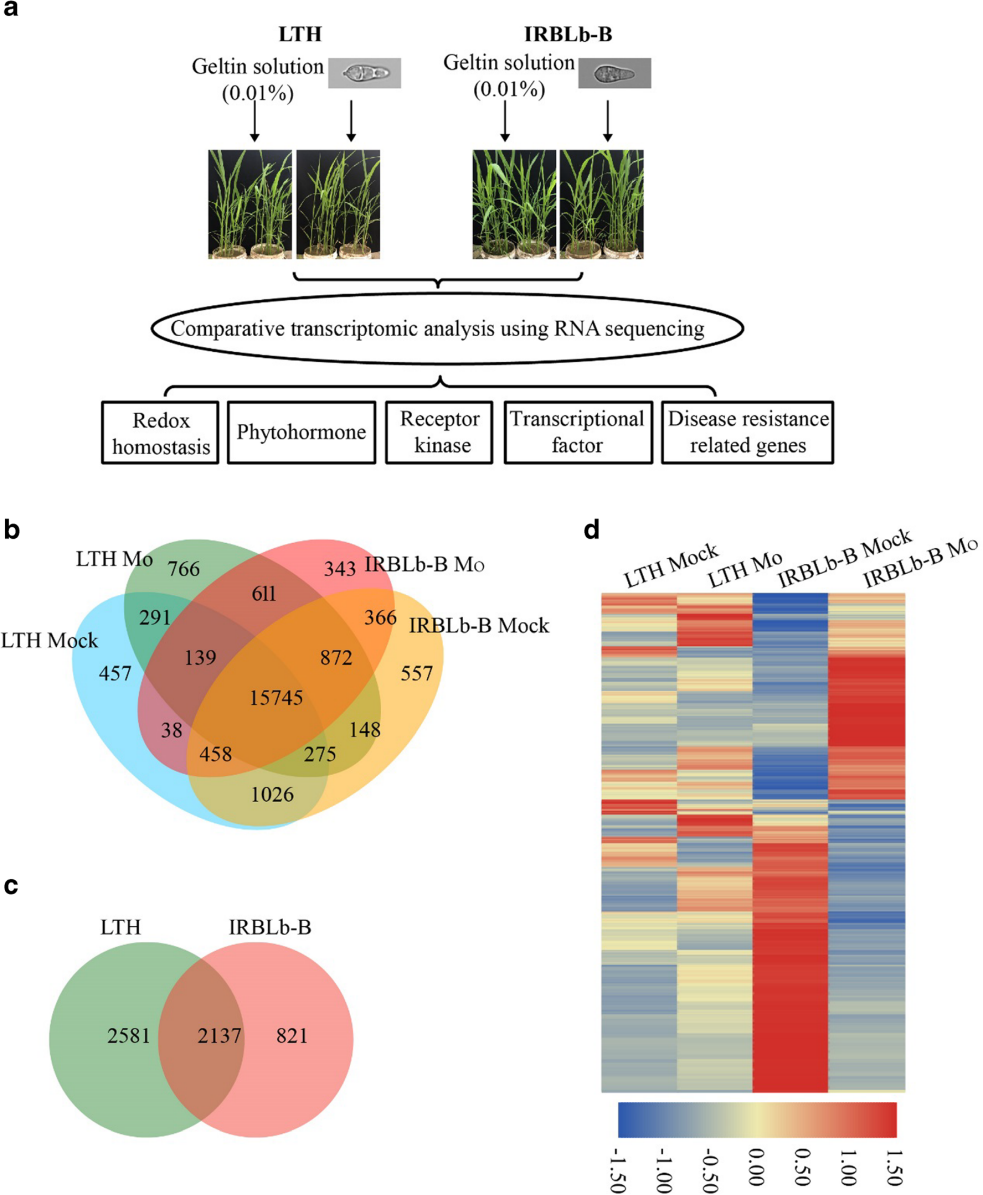
The RNA-seq workflow and global profiling. **a** The RNA-seq workflow of IRBLb-B and LTH with *M. oryzae* strain P131 infection. Redox homeostasis, phytohormone, receptor kinase, transcriptional factor, and disease resistance related genes were analyzed to reveal the *Pib*-mediated blast resistance. **b** The RNA-seq based identification of differential transcription in seedlings of IRBLb-B and LTH sampled 36 hpi with *M. oryzae* strain P131. Venn diagram illustrating the number of genes detected present in both genotypes and/or treatments, and those present in specific genotype and/or treatment combinations. **c** The venn diagram of SDEGs (FDR adjusted *p* ≤ 0.05 and absolute log_2_ fold ≥ 2) across LTH and IRBLb-B. **d** The heatmap of SDEGs across LTH and IRBLb-B

To further investigate the mechanism of *Pib*-mediated rice blast resistance, the strategy employed to acquire the transcriptomes of *M. oryzae*-inoculated seedlings is illustrated in Fig. 2a. Approximately 5 × 10^6^ clean reads were obtained from each biological replicate (Table S2), over 95% of which were successfully aligned with the cv. Nipponbare genome sequence. A Pearson correlation analysis conducted between the biological replicates revealed strong positive coefficients for both two genotypes (Tables S3, S4). The number of genes which were not transcriptionally altered by *M. oryzae* infection and were represented in both IRBLb-B and LTH was 15,745 (Fig. 2b). The number of SDEGs identified in LTH was 4718, of which 2286 were up-regulated by *M. oryzae* infection and 2432 were down-regulated; the equivalent numbers in IRBLb-B were 2958 SDEGs (815 up- and 2143 down-regulated) (Figs. 2c and S1). Among the two sets of SDEGs, 2137 (588 up- and 1549 down-regulated) were shared between LTH and IRBLb-B (Fig. 2c). The numbers of two genotype-specific SDEGs were 2581 (LTH) and 821 (IRBLb-B) (Fig. 2c, d).

### qRT-PCR validation of differential transcription deduced from the RNA-seq data

**Fig. 3:**
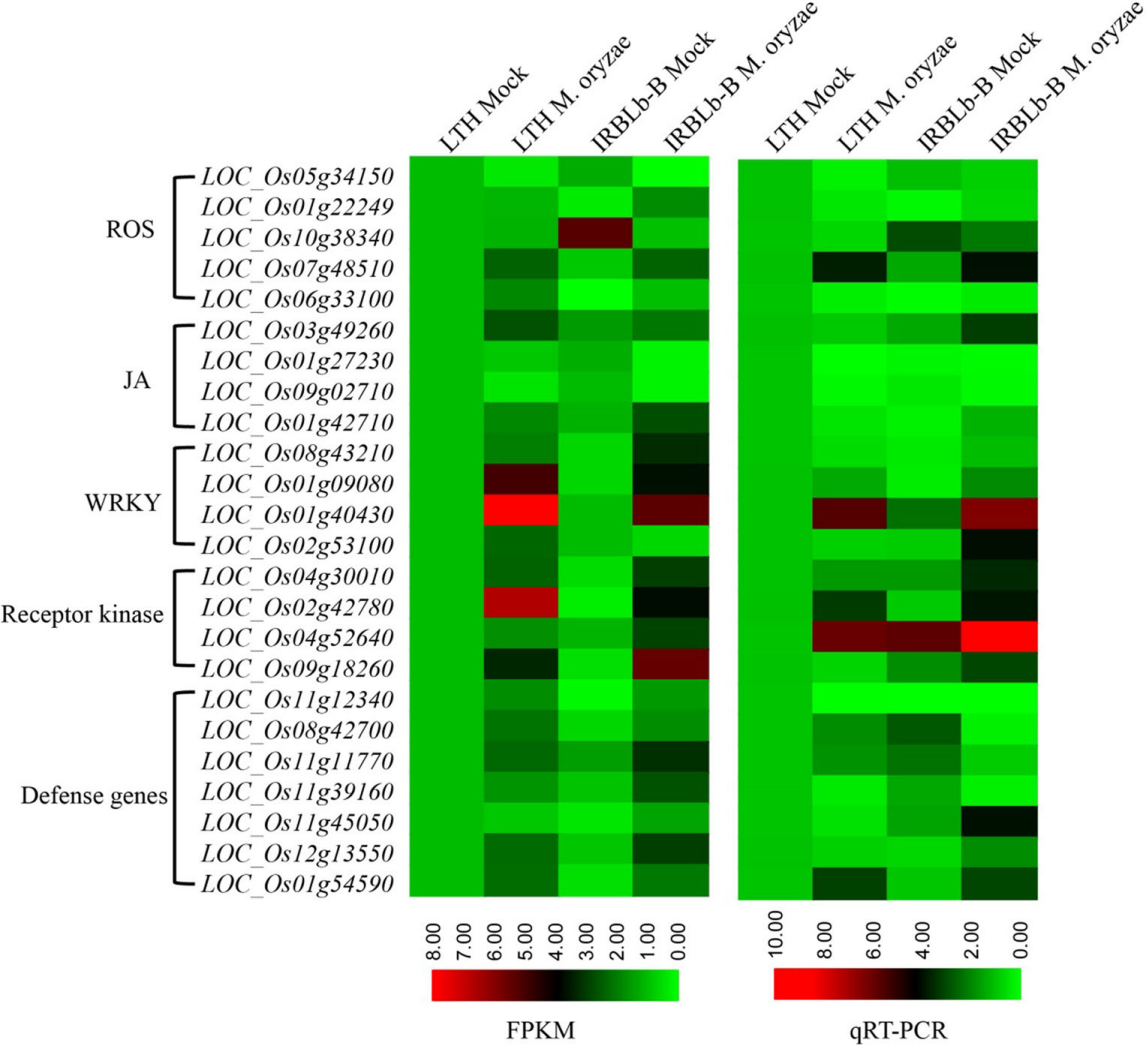
Validation of RNA-seq based on identification of differential transcription using qRT-PCR. Twenty-four SDEGs, including ROS, JA, WRKY, Receptor kinase, and defense related genes, were selected for validation. The heatmap was carried out by TBtools software based on FPKM and 2^−ΔΔCT^ data. *Tubulin* gene (*LOC_Os03g13170*) was chosen as the reference sequence. The LTH Mock as the reference sample

In order to verify the results obtained by RNA-seq analysis, a sample of 24 IRBLb-B specific *M. oryzae*-induced SDEGs, all experiencing a fold change in transcript abundance of at least four (Table S5), was used to validate the RNA-seq data using qRT-PCR. This set of SDEGs included five ROS-related genes, four JA-related genes, four WRKY transcription factors, four receptor kinase, and seven disease-resistant related genes. The outcome of the qRT-PCR assay was almost same pattern with the conclusion based on the RNA-seq data (Fig. 3; Table S5, Table S14).

### GO enrichment and KEGG pathway analysis of the SDEGs

**Fig. 4:**
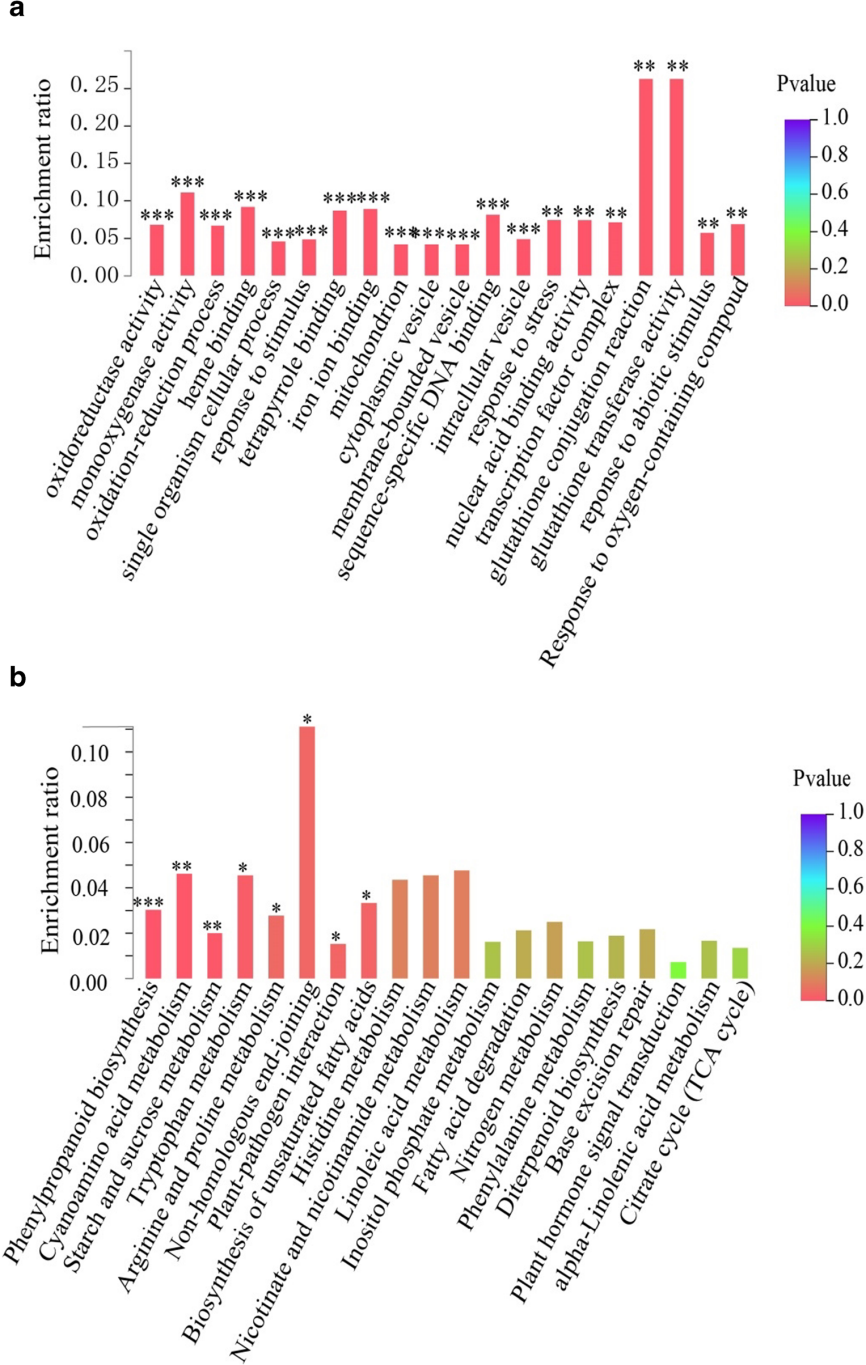
GO and KEGG enriched analysis of the 821 SDEGs detected uniquely in *M. oryzae*-inoculated IRBLb-B. **a** The various cellular components, biological processes, and molecular functions identified by GO enriched analysis. **b** KEGG enrichment analysis. The significance of enrichment with the *P* ≤ 0.05, 0.01, and 0.001 are marked by *, **, and ***, respectively

To explore signaling pathways involved in the *Pib*-mediated blast resistance, a summary of the GO analysis of the 821 *M. oryzae*-infection induced IRBLb-B SDEGs (Table S6, S7): the enriched GO terms related to 28 molecular functions, seven cellular components, and 41 biological processes. The GO analysis showed that most enriched GO terms were related to redox balance, including oxidoreductase activity, monooxygenase activity, oxidation-reduction process, glutathione conjugation reaction, glutathione transferase activity, and response to oxygen-containing compound. Moreover, stress response GO terms, including response to stimulus, response to stress, and response to abiotic stimulus, were enriched. These results indicated that redox balance and stress response-related genes may be involved in *Pib*-mediated blast resistance (Fig. 4a). In order to analyze metabolic pathways involved in the *Pib*-mediated blast resistance, a KEGG pathway analysis identified that both primary (including starch and sucrose metabolism, amino acid metabolism, and fatty acid metabolism) and secondary (phenylpropanoid synthesis) metabolism, as well as plant–pathogen interactions, were enriched (Fig. 4b; Table S8). To further elucidate the different pathways and the genes in them that get induced in the resistant and susceptible lines, a KEGG analysis was carried out and found that the secondary metabolites, plant hormone signal transduction, MAPK signaling pathway, and plant-pathogen interaction were enriched in both resistant and susceptible lines. Peroxisomes and some primary metabolism including carbon metabolism and nitrogen metabolism were specifically enriched in resistant lines, suggesting that energy metabolism and peroxisome involved in resistance of IRBLb-B to rice blast (Table S13).

### Redox homeostasis

**Fig. 5:**
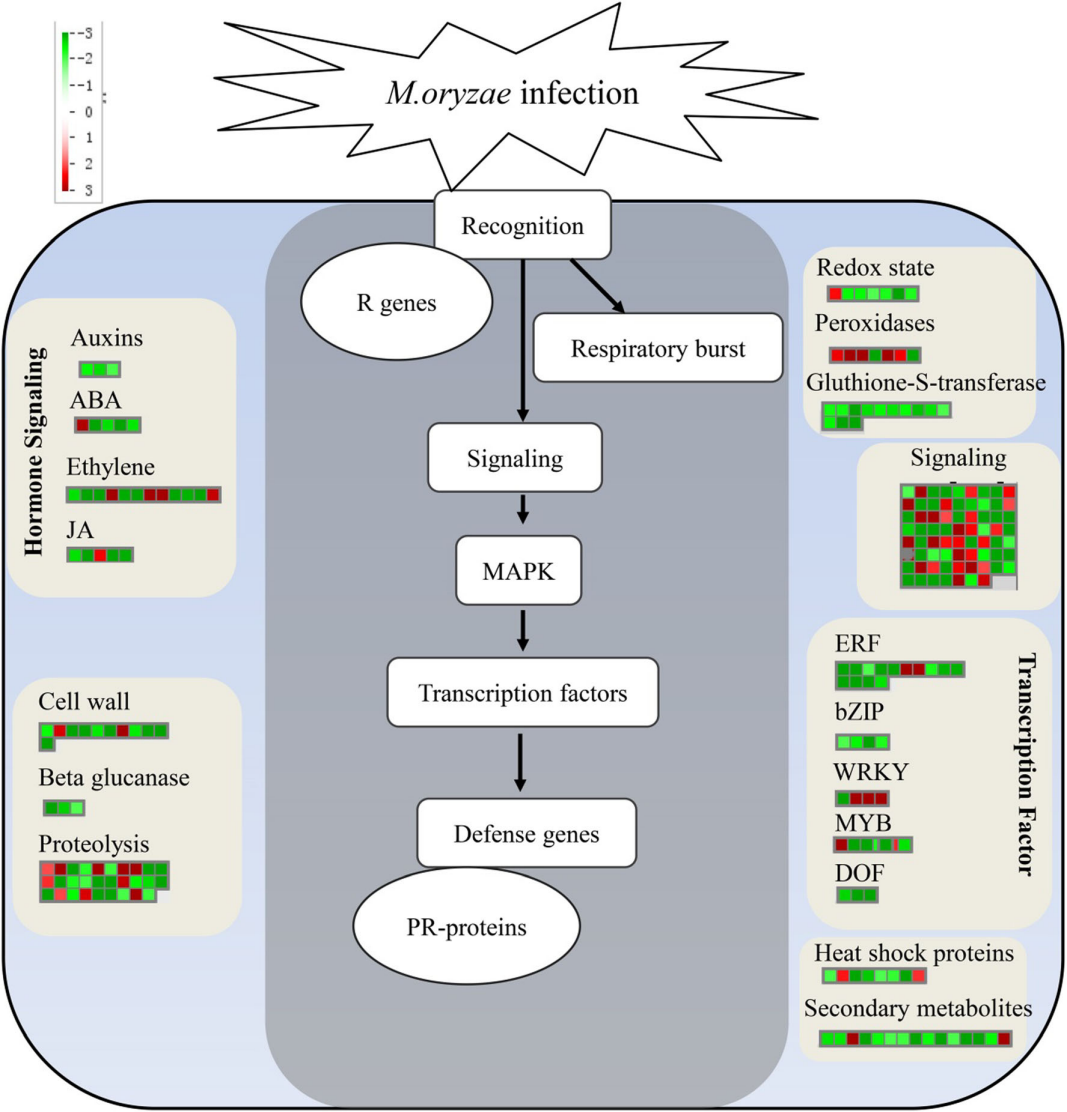
MapMan overview of the SDEGs, unique to IRBLb-B upon *M. oryzae* infection, involved in biotic stress pathway. Values represent log_2_ fold changes. Red cells indicate up-regulated genes and green cells down-regulated genes. ABA abscisic acid, JA jasmonate, ERF ethylene response factor, bZIP basic region-leucine zipper, DOF DNA binding with one finger, MAPK mitogen-activated protein kinase. PR-protein pathogenesis-related protein

ROS (such as superoxide, hydrogen peroxide, hydroxyl radical, and singlet oxygen) is the earliest and rapid defense response to limit *M. oryzae* spread (Kou et al. 2019; Lehmann et al. 2015). ROS were fine-tuned by several enzymes, including NADPH oxidase and scavenging enzymes (redoxin, peroxidase, catalase, and glutathioneredoxin) (Camejo et al. 2016). Our study showed that genes encoding three categories of respiratory burst proteins (redox state, peroxidases, and glutathione S-transferases) were represented among the set of IRBLb-B specific *M. oryzae*-induced SDEGs (Fig. 5; Table S9). Seven of these (five up-regulated by *M. oryzae* infection and two down-regulated) encoded peroxidase precursors. Five of the SDEGs encoded ROS scavenging compounds, namely, thioredoxin (*LOC_Os07g48510*), glutaredoxin (*LOC_Os01g47760*, *LOC_Os02g51370*, and *LOC_Os05g48930*), and peroxiredoxin (*LOC_Os06g09610*), all of which down-regulated by *M. oryzae* infection. Fourteen of the SDEGs (all down-regulated by *M. oryzae* infection) encoded a glutathione S-transferase. These result showed that redox homeostasis–related genes were modulated in IRBLb-B with rice blast infection.

**Fig. 6:**
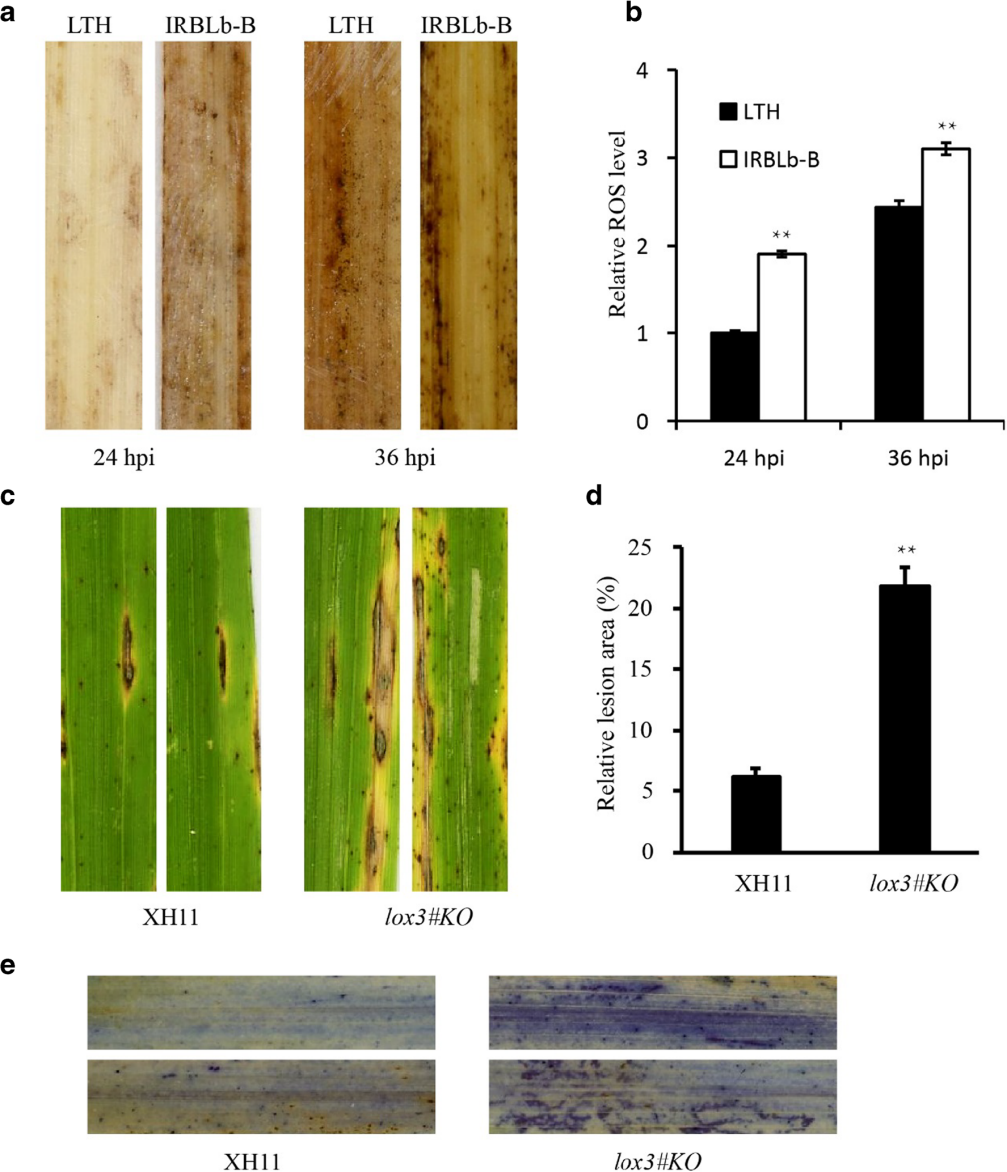
DAB staining analysis of LTH and IRBLb-B with *M. oryzae* infection at 24 hpi and 36 hpi and the phenotype of lesions on WT XH11 and *lox3* knockout mutant inoculation by enshi2-2 strain. **a** DAB stain showed that IRBLb-B accumulated a relative high level of hydrogen peroxide compared with LTH at 24 hpi and 36 hpi. **b** The relative hydrogen peroxide level by ImageJ software, the LTH 24 hpi as the reference. The error bar represents the standard deviation for triplicate assays. **c** Leaf phenotype of XH11 and lox3#KO after inoculation by enshi2-2 strain. **d** The relative lesion area of XH11 and lox3#KO after inoculation by enshi2-2 strain. **e** The trypan blue stain of XH11 and lox3#KO after inoculation by enshi2-2 strain

Next, we determined the ROS accumulation in IRBLb-B with rice blast infection. DAB was used to stain the infected leaves of LTH and IRBLb-B to detect the production of hydrogen peroxide. As shown in Fig. 6, no DAB staining was observed in LTH leaves with *M. oryzae* infection at 24 hpi, while obvious DAB staining was observed in that of IRBLb-B. By 36 hpi, both LTH and IRBLb-B leaves showed obvious DAB staining, while the leaves of IRBLb showed more and clear DAB staining (Fig. 6b). These results indicated that the resistance of IRBLb-B to rice blast was related to the accumulation of more hydrogen peroxide.

### Phytohormone-related genes

Plant hormones play important roles in response to biotic stress (Spoel and Dong, 2008). The set of IRBLb-B specific *M. oryzae*-induced SDEGs included four down-regulated genes associated with JA metabolism, namely, two encoding a 12-oxophytodienoate reductase (*LOC_Os01g27230* and *LOC_Os06g11280*), one a lipoxygenase (*LOC_Os12g37320*) and one a CBS domain-containing membrane protein (*LOC_Os09g02710*), along with one up-regulated, encoding a lipoxygenase (*LOC_Os03g49260*) (Figs. 5 and 6; Table S10). *Oslox3#KO* was more susceptible to rice blast, suggesting that *LOX3* played a positive regulatory role in rice blast resistance. A further twelve of the SDEGs were associated with ethylene (ET)-mediated processes: seven (three up- and four down-regulated) encoded AP2/ERF domain-containing proteins, one (down-regulated) ET synthesis-related gene (*LOC_Os08g30080*), and four (three up- and one down-regulated) genes (*LOC_Os09g02710*, *LOC_Os10g31240*, *LOC_Os05g41760*, and *LOC_Os12g36630*) regulated by ET. Three of the SDEGs encoded auxin metabolism products: these comprised two auxin synthesis-related proteins and one protein regulated by auxin. Four genes associated with abscisic acid (ABA) metabolism were identified as IRBLb-B specific *M. oryzae*-induced SDEGs (all were down-regulated): three encoded proteins related with ABA synthesis and one a protein regulated by ABA.

### Kinase-mediated signaling-related genes

Pathogen signal perception and activation of downstream defense signaling molecules, including receptor kinases (RKs), MAPK, and calmodulin-dependent calcium sensor protein, are vital for plant defense (Jain et al. 2017). We determined whether RKs are involved in defense response against *M. oryzae* in IRBLb-B. In all, thirty-seven genes encoding a receptor kinase were classified as IRBLb-B specific *M. oryzae*-induced SDEGs (eighteen were up- and nineteen down-regulated by *M. oryzae* infection); this set included fourteen genes encoding members of the leucine-rich receptor subfamily (eleven up-regulated by *M. oryzae* infection), four members of the leucine-rich kinase 10L subfamily (one up-regulated by *M. oryzae* infection), nine members of the lectin-like receptor kinase subfamily (four up-regulated by *M. oryzae* infection), five members of the S-domain receptor-like protein kinase subfamily (one up-regulated by *M. oryzae* infection), and five members of the cell wall-associated kinase subfamily (one up-regulated by *M. oryzae* infection) (Fig. 5; Table S11). In addition, four genes encoding a calmodulin-dependent protein were down-regulated in response to *M. oryzae* infection, and two encoding a G protein were different-regulated in response to *M. oryzae* infection.

### Transcription factor–related genes

It is well known that transcription factors, such as WRKY, MYB, and AP2/ERF, play important roles in response to pathogens in plant (Amorim et al. 2017). A total of twenty eight of the IRBLb-B specific *M. oryzae*-induced SDEGs encoded a transcription factor; these included *WRKY26*, *WRKY32*, and *WRKY17* (which were all up-regulated by *M. oryzae* infection) and *WRKY100* (down-regulated); *MYB106*, *MYB103L*, *ARM1*, and *MYB7* (which were all up-regulated); and *MYB61* (down-regulated). A further fifteen SDEGs (three up- and twelve down-regulated) encoded members of the AP2/ERF family, along with four (three up- and one down-regulated) encoding members of the bHLH family (Fig. 5; Table S12). Collectively, these results indicated that WRKY, MYB, AP2/ERF, and bHLH transcription factors may be involved in signal transduction of *Pib* to regulate the expression of defense genes in IRBLb-B.

### Disease resistance–related genes

To investigate disease resistance–related genes related to *M. oryzae* infection in IRBLb-B, we searched for resistance-related genes in SDEGs unique to IRBLb-B. Seven putative disease resistance–related genes were among the set of IRBLb-B specific *M. oryzae*-induced SDEGs: these included *Pib* itself, three encoding a type of NBS-LRR protein (*LOC_Os11g39160*, *LOC_Os11g45050*, and *LOC_Os12g13550*), two *PRM1* genes (*LOC_Os11g11770* and *LOC_Os11g12340*), and one encoding a resistance-associated protein (*LOC_Os08g42700*) (Table 1). The abundance of *Pib* transcript was 4.3 fold higher in *M. oryzae*-infected than in mock-inoculated IRBLb-B plants (Table S6).

**Table 1:** Disease resistance–related genes identified in IRBLb-B with *M. oryzae* infection

Gene_ID	IRBLb-B	LTH	Annotation
	FC (*M. oryzae*/mock)	FC (*M. oryzae*/mock)	
LOC_Os02g57310	4.353	0.499	Pib, putative, expressed
LOC_Os08g42700	4.067	3.11	Resistance protein, putative, expressed
LOC_Os11g11770	4.519	3.755	Disease resistance protein RPM1, putative, expressed
LOC_Os11g12340	11.503	2.427	Disease resistance protein RPM1, putative, expressed
LOC_Os11g39160	4.942	1.872	NBS-LRR disease resistance protein, putative
LOC_Os11g45050	4.662	0.85	NBS-LRR disease resistance protein, putative, expressed
LOC_Os12g13550	7.27	3.645	NBS-LRR disease resistance protein, putative, expressed

## Discussion

Characterization of the rice-*M. oryzae* interaction has succeeded in detecting the activity of a number of *R* genes (Meng et al. 2019; Wang et al. 2017). Here, an attempt was made to reveal the mechanistic basis of *Pib*-mediated resistance by comparing the RNA-seq-derived transcriptomes of *M. oryzae*-inoculated LTH and its *Pib* NIL IRBLb-B. The number of genes in LTH which were differentially transcribed in the blast susceptible cultivar LTH as a result of *M. oryzae* infection was 4718, while a smaller number (2958) was induced in the blast resistant IRBLb-B plants. A further analysis of the latter set of genes suggested that a substantial number of them encoded redox homeostasis, WRKY transcription factors, and receptor kinases, along with proteins involved in either phytohormone metabolism or defense signaling. To our surprise, more than half of the genes were down-regulated upon *M. oryzae* infection in IRBLb-B. There may be some antagonism between growth and disease resistance, and plants that exhibit better resistance often sacrifice certain growth (Jiang et al. 2017).

The synthesis of ROS is an important component of the early phase of the defense mounted by rice against *M. oryzae* (Lehmann et al. 2015). These compounds act both to strengthen the plant’s cell walls and/or to trigger a number of downstream defense genes (Dangol et al. 2018; Gadjev et al. 2006). ROS typically accumulate to a higher level when the host and the pathogen are incompatible (Gupta et al. 2012; Jain et al. 2017). Here, the ROS accumulation in IRBLb-B plants infected with the avirulent *M. oryzae* strain P131 was higher than that in LTH (Fig. 6), and the ROS level was also accumulated in LTH at 36 hpi compared with 24 hpi, which indicated that ROS participated in the basic resistant of compatibility and incompatibility. Maintaining ROS content is likely basic requirement for *Pib*-mediated resistance.

Phytohormones, especially salicylic acid (SA), JA, and ET, are intimately involved in the host’s response to pathogen invasion (Pieterse et al. 2009; Spoel et al. 2003). JA and ethylene-related genes were induced during *M. oryzae* infection, while most of gene-related JA or ethylene were down-regulated in IRBLb-B, this may be due to the different sampling time. Some related genes were up-regulated in the early stage of infection and down-regulated in the late stage of infection. For example, *OsLOX2*, *OsLOX5*, *OsCOL1b*, and *OsCOL2* show this expression pattern (Zhang et al. 2018a). After knock out the gene *LOX3* which is significant induced by rice blast in IRBLb-B, we found that *lox3#KO* was more susceptible to rice blast, which indicated that the resistance of IRBLb-B required the participation of JA-related genes. More recently, other plant hormones, including ABA and auxin have also emerged as crucial regulator of plant–pathogen interactions (Anderson et al. 2004; Jiang et al. 2017; Jiang et al. 2013; Yazawa et al. 2012). In this study, three genes encoding auxin metabolism products and five IRBLb-B SDEGs encoding products associated with ABA metabolism were found differentially expressed in IRBLb-B and LTH. Overall, it is suggested that at least four kinds of phytohormones participate in *Pib*-mediated resistance to rice blast.

Pathogen signal perception, followed by the activation of downstream defense signaling molecules (receptor kinases, mitogen-activated protein kinases, and calmodulin-dependent calcium sensor proteins), is a fundamental part of the plant defense response (Jain et al. 2017). An analysis of the durably *M. oryzae*-resistant rice cultivar Digu has revealed many examples of the differential transcription of receptor kinase-encoding genes (Li et al. 2016), as is also the case for IRBL18, IRBL22 (Wei et al. 2013), and PB1+*Pi9* (Jain et al. 2017). Here, thirty-seven receptor kinase encoding genes were present among the set of IRBLb-B SDEGs responding to *M. oryzae* infection. Some transcription factors have been reported to play important roles during perception of pathogen infection by receptor kinase. In particular, *WRKY*, *MYB*, and *AP2/ERF* genes can all be triggered when plants are exposed to a pathogen (Amorim et al. 2017; Dong et al. 2003; Fernandez-Calvo et al. 2011; Singh et al. 2002; Wiermer et al. 2005). In the present study, the set of IRBLb-B SDEGs responding to *M. oryzae* infection included four genes encoding WRKY factors, five MYB factors, fifteen AP2/ERF factors, and four bHLH factors, underlining the importance role of these transcription factors in signal transduction during the *Pib*-mediated defense response.

**Fig. 7:**
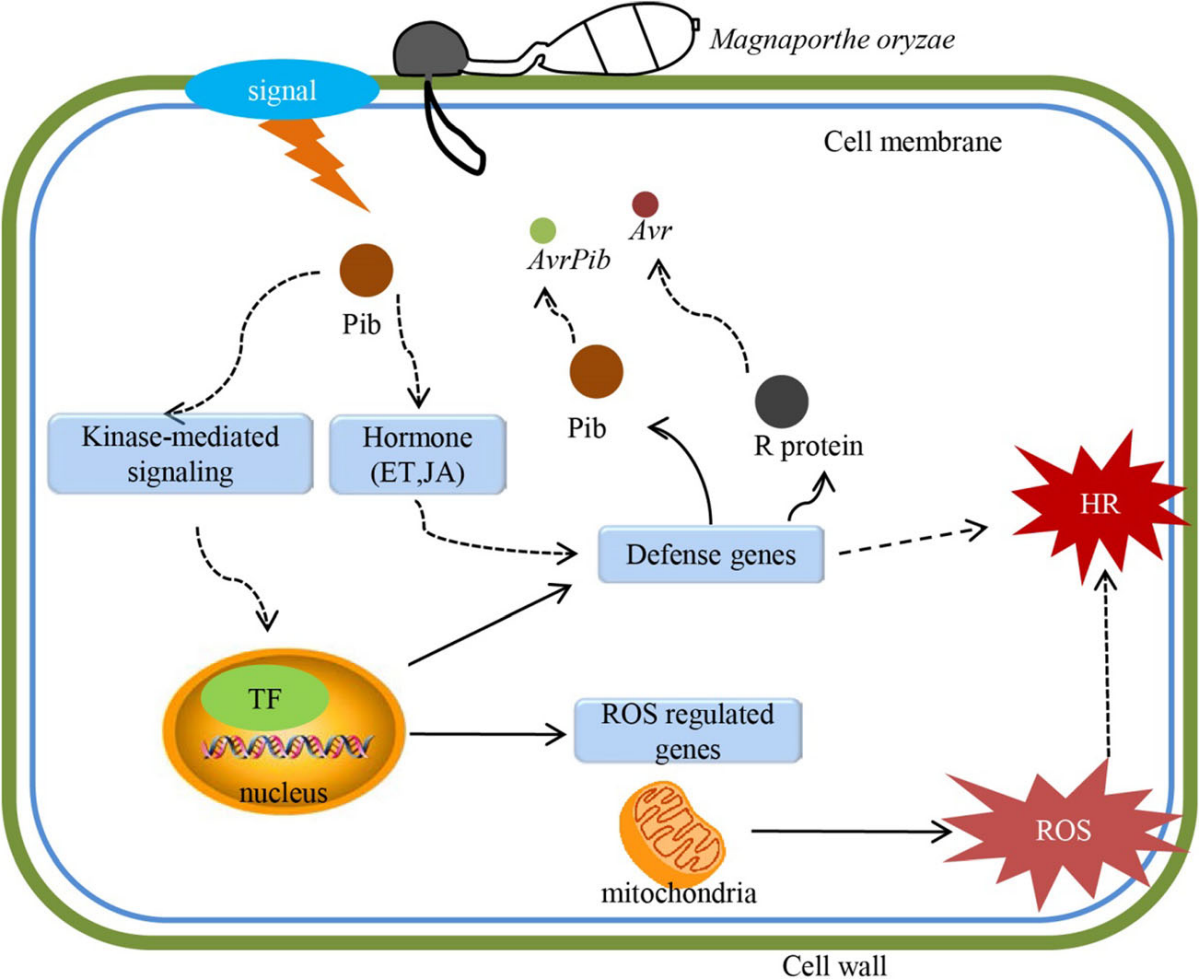
A putative mechanistic model of the broad spectrum resistance mediated by *Pib*. The fungal avirulence factor is recognized by the Pib protein, which triggers kinase-mediated and hormone signaling to transduce the appropriate signal to a suite of downstream transcription factors. The activation of these transcription factors regulates genes encoding products involved in ROS metabolism and various defense-related proteins, leading eventually to the expression of the hypersensitive response. ET ethylene, JA jasmonate, TF transcription factor, ROS reactive oxygen species, HR hypersensitive response

Plants dynamically reprogram their transcriptome upon pathogen attack (Pandey and Somssich 2009). Here, it has been shown that the plants harboring the broad spectrum blast resistance gene *Pib* activated a cascade of defense-related genes when challenged with the pathogen (Fig. 3). A model of the *Pib*-mediated defense response was given in Fig. 7. Upon exposure to *M. oryzae*, *Pib* is induced, resulting in the activation of a suite of downstream genes following signal transduction via both kinase-mediated and phytohormone-interacting pathways. Various transcription factors function to regulate the expression of key disease resistance–related and/or ROS-related genes.

## Conclusions

Here, the transcriptomes of LTH and its NIL IRBLb-B have been compared, specifically with a view to reveal the transcriptional impact of the presence of the rice blast pathogen. The analysis identified that 821 genes were significant differentially transcribed in IRBLb-B when challenged by an incompatible race of *M. oryzae*. Many of these gene-encoded products are involved in ROS metabolism, phytohormone-mediated signaling, transcription factors-mediated signaling, and kinase-mediated signaling. The set of IRBLb-B SDEGs responding to *M. oryzae* infection included seven disease resistance–related genes. Thus, this study revealed that IRBLb-B plants were able to modulate some important candidate genes transcriptomic reprogramming upon *M. oryzae* attack.

## Electronic supplementary material

ESM 1 (XLSX 13 kb)

ESM 2 (DOCX 17 kb)

ESM 3 (DOCX 16 kb)

ESM 4 (DOCX 16 kb)

ESM 5 (XLSX 10 kb)

ESM 6 (XLSX 214 kb)

ESM 7 (XLSX 30 kb)

ESM 8 (XLSX 14 kb)

ESM 9 (XLSX 11 kb)

ESM 10 (XLSX 11 kb)

ESM 11 (XLS 37 kb)

ESM 12 (XLSX 12 kb)

ESM 13 (XLSX 11 kb)

ESM 14 (XLSX 11 kb)

Fig. S1 Volcano plot illustrating genes which were differentially transcribed in both LTH and IRBLb-B as a result of *M. oryzae* infection (JPG 1175 kb)
