# Supplementary material for: Comparative transcriptomic analysis reveals the mechanistic basis of Pib-mediated broad spectrum resistance against Magnaporthe oryzae
Source: Funct Integr Genomics. 2020 Sep 7;20(6):787–99. doi: 10.1007/s10142-020-00752-x (PMC7585573; doi:10.1007/s10142-020-00752-x)
Supplement: Supplementary file 2 — (DOCX 17 kb) [file 10142_2020_752_MOESM2_ESM.docx]

Table S2. Mapping the RNA-seq reads obtained from LTH and IRBLb-B onto the rice (cv. Nipponbare) genome sequence. R: biological replicate.

| Rice Lines | Total reads | Total mapped | Multiple mapped | Uniquely mapped |
| --- | --- | --- | --- | --- |
| Susceptible Line |  |  |  |  |
| Mock LHT R1 | 51394716 | 49603630(96.52%) | 2444586(4.76%) | 47159044(91.76%) |
| Mock LHT R2 | 48057758 | 46146224(96.02%) | 2143722(4.46%) | 44002502(91.56%) |
| Mock LHT R3 | 52970366 | 50987333(96.26%) | 3866006(7.3%) | 47121327(88.96%) |
| *M. oryzae* LHT R1 | 45574574 | 43457383(95.35%) | 4877770(10.7%) | 38579613(84.65%) |
| *M. oryzae* LHT R2 | 47422452 | 44891401(94.66%) | 7348934(15.5%) | 37542467(79.17%) |
| *M. oryzae* LHT R3 | 47735620 | 45623015(95.57%) | 7110330(14.9%) | 38512685(80.68%) |
| Resistant Line |  |  |  |  |
| Mock *Pib* R1 | 48696490 | 46805744(96.12%) | 3176585(6.52%) | 43629159(89.59%) |
| Mock *Pib* R2 | 50025294 | 48215031(96.38%) | 6950997(13.89%) | 41264034(82.49%) |
| Mock *Pib* R3 | 49032620 | 47369447(96.61%) | 8246983(16.82%) | 39122464(79.79%) |
| *M. oryzae* *Pib* R1 | 44370398 | 42524382(95.84%) | 2532027(5.71%) | 39992355(90.13%) |
| *M. oryzae* *Pib* R2 | 41507552 | 40011944(96.4%) | 3537229(8.52%) | 36474715(87.87%) |
| *M. oryzae* *Pib* R3 | 47756718 | 45918470(96.15%) | 8058017(16.87%) | 37860453(79.28%) |
