# Supplementary material for: Comparative transcriptomic analysis reveals the mechanistic basis of Pib-mediated broad spectrum resistance against Magnaporthe oryzae
Source: Funct Integr Genomics. 2020 Sep 7;20(6):787–99. doi: 10.1007/s10142-020-00752-x (PMC7585573; doi:10.1007/s10142-020-00752-x)
Supplement: Supplementary file 3 — (DOCX 16 kb) [file 10142_2020_752_MOESM3_ESM.docx]

Table S3. Pearson correlation coefficients calculated between biological replicates of LTH either infected with *M. oryzae* or mock inoculated. R: biological replicate.

| LHT | *M. oryzae* LHT R1 | Mock LHT R3 | *M. oryzae* LHT R3 | Mock LHT R1 | *M. oryzae* LHT R2 | Mock LHT R2 |
| --- | --- | --- | --- | --- | --- | --- |
| *M. oryzae* LHT R1 | 1 | 0.869 | 0.756 | 0.707 | 0.879 | 0.819 |
| Mock LHT R3 | 0.869 | 1 | 0.626 | 0.608 | 0.75 | 0.852 |
| *M. oryzae* LHT R3 | 0.756 | 0.626 | 1 | 0.901 | 0.908 | 0.868 |
| Mock LHT R1 | 0.707 | 0.608 | 0.901 | 1 | 0.893 | 0.903 |
| *M. oryzae* LHT R2 | 0.879 | 0.75 | 0.908 | 0.893 | 1 | 0.896 |
| Mock LHT R2 | 0.819 | 0.852 | 0.868 | 0.903 | 0.896 | 1 |
