# Supplementary material for: Comparative transcriptomic analysis reveals the mechanistic basis of Pib-mediated broad spectrum resistance against Magnaporthe oryzae
Source: Funct Integr Genomics. 2020 Sep 7;20(6):787–99. doi: 10.1007/s10142-020-00752-x (PMC7585573; doi:10.1007/s10142-020-00752-x)
Supplement: Supplementary file 4 — (DOCX 16 kb) [file 10142_2020_752_MOESM4_ESM.docx]

Table S4. Pearson correlation coefficients calculated between biological replicates of IRBLb-B either infected with *M. oryzae* or mock inoculated. R: biological replicate.

| *Pib* | Mock *Pib* R1 | *M.oryzae* *Pib* R1 | Mock *Pib* R2 | *M.oryzae* *Pib* R2 | Mock *Pib* R3 | *M.oryzae* *Pib* R3 |
| --- | --- | --- | --- | --- | --- | --- |
| Mock *Pib* R1 | 1 | 0.81 | 0.729 | 0.854 | 0.861 | 0.778 |
| *M.oryzae* *Pib* R1 | 0.81 | 1 | 0.667 | 0.902 | 0.801 | 0.717 |
| Mock *Pib* R2 | 0.729 | 0.667 | 1 | 0.807 | 0.886 | 0.915 |
| *M.oryzae Pib* R2 | 0.854 | 0.902 | 0.807 | 1 | 0.922 | 0.898 |
| Mock *Pib* R3 | 0.861 | 0.801 | 0.886 | 0.922 | 1 | 0.936 |
| *M.oryzae* *Pib* R3 | 0.778 | 0.717 | 0.915 | 0.898 | 0.936 | 1 |
