# Supplementary figures and images for: Comparative transcriptomic analysis reveals the mechanistic basis of Pib-mediated broad spectrum resistance against Magnaporthe oryzae
Source: Funct Integr Genomics. 2020 Sep 7;20(6):787–99. doi: 10.1007/s10142-020-00752-x (PMC7585573; doi:10.1007/s10142-020-00752-x)

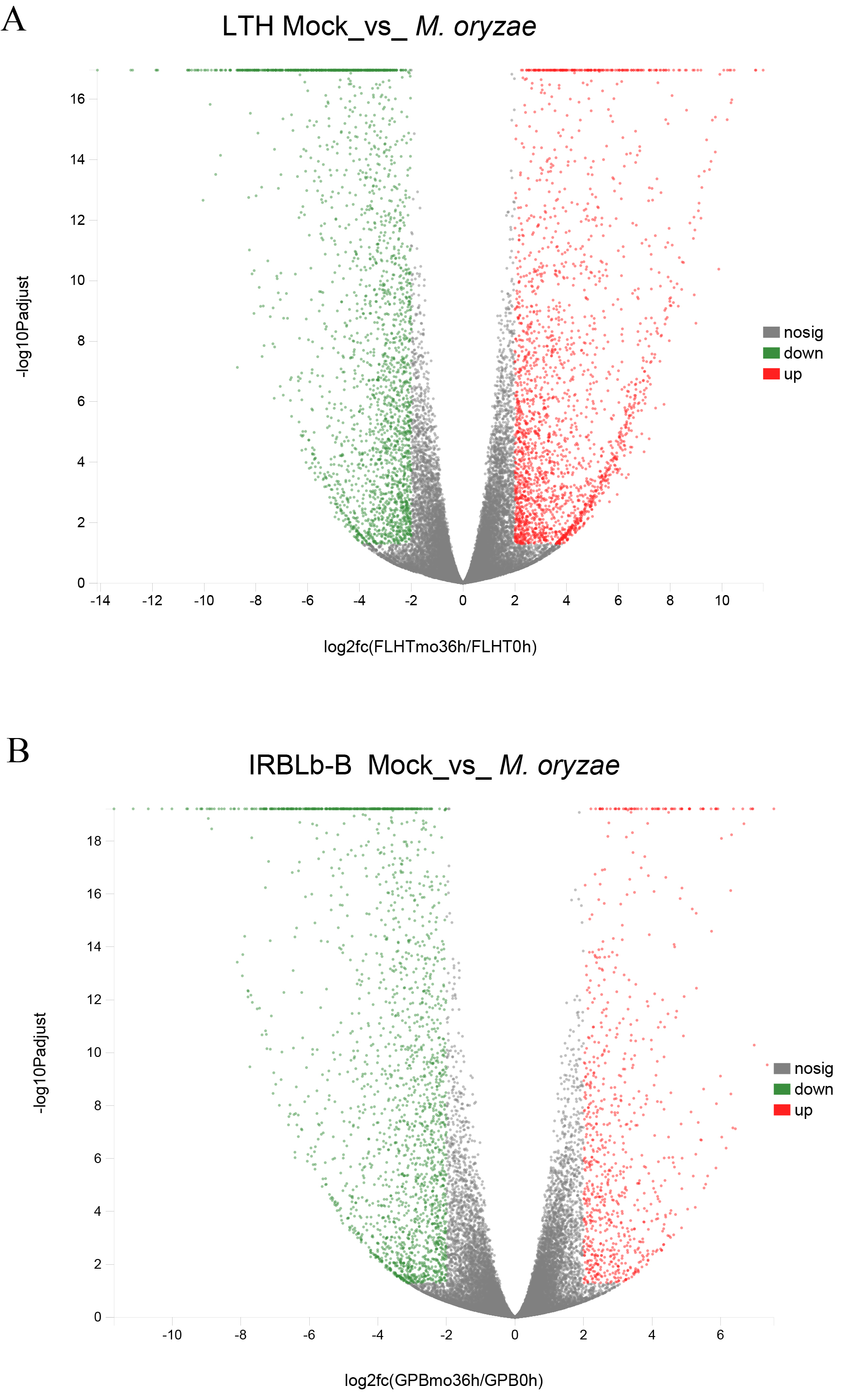

Supplement: Supplementary file 15 — Volcano plot illustrating genes which were differentially transcribed in both LTH and IRBLb-B as a result of M. oryzae infection (JPG 1175 kb) [file 10142_2020_752_MOESM15_ESM.jpg]
